# Lynch Syndrome in Thai Endometrial Cancer Patients

**DOI:** 10.31557/APJCP.2021.22.5.1477

**Published:** 2021-05

**Authors:** Tarinee Manchana, Chai Ariyasriwatana, Surang Triratanachat, Prasit Phowthongkum

**Affiliations:** 1 *Division of Gynecologic Oncology, Department of Obstetrics and Gynecology, Faculty of Medicine, Chulalongkorn University and King Chulalongkorn Memorial Hospital, Bangkok, Thailand. *; 2 *Division of Gynecologic Pathology and Cytology, Faculty of Medicine, Chulalongkorn University and King Chulalongkorn Memorial Hospital, Bangkok, Thailand. *; 3 *Division of Medical Genetics, Department of Medicine, Faculty of Medicine, Chulalongkorn University and King Chulalongkorn Memorial Hospital, Bangkok, Thailand.*

**Keywords:** Endometrial cancer, hereditary nonpolyposis colorectal cancer, immunohistochemistry, lynch syndrome

## Abstract

**Background:**

Lynch syndrome increases lifetime risk of endometrial cancer to 40-60%. Screening with molecular tumor testing for mismatch repair (MMR) proteins have been recommended. This study aims to evaluate the incidence of MMR deficiency and germline mutation in endometrial cancer Thai patients.

**Methods:**

Immunohistochemistry for MMR proteins, including MLH1, MSH2, MSH6 and PMS2 were tested in 166 surgical specimens. Patients who had MMR deficiencies were offered genetic counseling and a germline testing using gene-panel next generation sequencing.

**Results:**

Fifty-eight of 166 patients (34.9%) had one or more MMR deficiencies which were: MLH1 and PMS2 in 42 patients (25.3%), MSH2 and MSH6 in 11 patients (6.6%), and MSH6 in 5 patients (3.0%). Of the 40 patients (24.1%) who met the revised Bethesda guidelines, 19 patients (47.5%) had MMR deficiency. In contrast, MMR deficiency was found in 39 of the 126 patients (31.0%) who did not meet the revised Bethesda guidelines. A total of 27 patients with MMR deficiencies agreed to have germline genetic testing. Germline MMR mutations were detected in 5 patients (18.5%) including *MSH6* (n=2), *PMS2* (n=2), and *MLH1* mutations (n=1). Incidental germline mutations in other genes were detected in 3 patients (1 *BRCA1*, 1 *PTEN*, and 1 *BARD1*). Among 5 Lynch syndrome patients, 2 patients (40%) did not meet the revised Bethesda guidelines. Eight patients who met the revised Bethesda Guidelines but having MMR proficiency had genetic testing, but no germline mutation was detected.

**Conclusion:**

MMR deficiencies were detected in 34.9% of the endometrial cancer patients. Germline mutations were diagnosed in 3.0% of this cohort (5/166 patients). Lynch syndrome screening with MMR immunohistochemistry should be considered in all patients regardless of personal or family history of Lynch syndrome-related cancers.

## Introduction

Hereditary endometrial cancer occurs in approximately 3-5% of all endometrial cancer patients. Lynch syndrome or hereditary nonpolyposis colorectal cancer (HNPCC) is the most common cause of hereditary endometrial cancer (Committee on Practice Bulletins-Gynecology and the Society of Gynecolgoic Oncology, 2014). Lynch syndrome is an autosomal dominant syndrome caused by genetic defects in one or more DNA mismatch repair (MMR) genes. This syndrome is associated with germline MMR gene mutations, including *MLH1, MSH2, MSH6, PMS2, *or *EPCAM* deletions, which may associate with epigenetic silencing of *MSH2* (Tamura et al., 2019). The incidence of MMR gene mutations in the general population is between 1:2000 and 1:660 (de la Chapelle, 2005). Lynch syndrome is classified as a predisposition to multiple types of cancers. The two most common Lynch syndrome-related cancers are colorectal and endometrial cancer. The lifetime risk for developing colorectal cancer increases from 4-5% in the general population to 40-60% in Lynch syndrome patients, and the lifetime risk of endometrial cancer increases from 3% to 40-60%. Other lifetime risks of cancers in Lynch syndrome include ovarian cancer (12%), stomach cancer (13%), small bowel cancer (5%), urinary tract cancer (4%), brain cancer (4%), and biliary tract cancer (2%) (Schmeler and Lu, 2008). Interestingly, the incidence of Lynch syndrome in endometrial cancer is slightly higher than in colorectal cancer, 1.4-5.9% (Watkins et al., 2017) and 0.7-3.6%, respectively (Hampel et al., 2005; Hampel et al., 2008; Pérez-Carbonell et al., 2012).

Approximately 15-20% of patients with epithelial ovarian, tubal and peritoneal cancer have *BRCA* mutations; therefore, all patients with these cancers should be offered *BRCA* germline testing (Randall et al., 2017). In contrast, Lynch syndrome is less prevalent; therefore, initial screening with clinical criteria, including personal and family history of Lynch syndrome-related cancers or molecular tumor testing is usually performed. The most common clinical criteria is the revised Bethesda Guidelines which are as follows; age at diagnosis below 50 years; synchronous or metachronous ovarian; colon or other Lynch syndrome-related cancers at any age; having a first degree relative with Lynch syndrome-related cancers being diagnosed before age 50 years old; or having two or more relatives with Lynch syndrome-related cancers at any age (Umar et al., 2004). Although the Revised Bethesda Guidelines remain the current clinical criteria for identifying individuals at risk for Lynch syndrome, these guidelines miss a large proportion of patients (Hampel et al., 2006). The Society of Gynecologic Oncology (SGO) and American College of Obstetricians and Gynecologists (ACOG) recommend the process of molecular evaluation of patients at risk for Lynch syndrome as follows: molecular tumor screening with immunohistochemistry for MMR genes expression and/or microsatellite instability followed by germline genetic testing if the screening test is positive (Randall et al., 2017).

MMR immunohistochemistry is sufficient for determining MMR deficiency in endometrial cancer since it has been reported to have a high concordance (94%) with microsatellite instability (Stelloo et al., 2017). Moreover, immunohistochemistry is less expensive and widely available especially in developing countries. Although, MMR deficiency testing is routinely performed in many countries, this screening test is not yet recommended in some countries including Thailand. This study aimed to evaluate the prevalence of MMR deficiency and germline MMR mutation in endometrial cancer Thai patients.

## Materials and Methods

This study was conducted on endometrial cancer patients who underwent primary surgery at the King Chulalongkorn Memorial Hospital, Bangkok, Thailand between January 2013 and January 2019. This study was approved by the Institutional Review Board, Faculty of Medicine, Chulalongkorn University. All study participants provided informed written consent prior to study enrollment. Patients with either endometrial cancer or synchronous endometrial and ovarian cancer were included. Patients who had no formalin fixed paraffin embedded block and incomplete medical records were excluded. The surgical specimens were reviewed by two gynecologic pathologists. MMR immunohistochemistry screening was performed on surgical specimens for 4 MMR proteins: MLH1, MSH2, MSH6, and PMS2. Normal MMR expression was defined as nuclear staining within tumor cells. The nuclear staining using nuclei of infiltrating lymphocytes and/or normal stromal cells were used as a positive internal control. Negative expression or loss of expression was defined as the complete absence of nuclear staining within tumor cells but presence of staining in normal endometrial and stromal cells. MMR deficiency was defined as negative or loss of expression of at least one of the four MMR proteins mentioned above and MMR proficiency was defined as intact MMR expression.

Demographic data, including age at diagnosis, parity, menopausal status, body mass index (BMI), family or personal history of cancer, pathological data, and previously received treatments, were retrieved from the medical records. Endometrial cancer patients were grouped according to the criteria of the revised Bethesda Guidelines. 

Patients with loss of MMR expression were offered genetic counseling by a geneticist. Germline testing was performed using DNA extracted from saliva or peripheral blood leukocytes. Full gene sequencing and deletion/duplication analyses were performed using the next-generation sequencing technology with 119 multi-gene panels associated with hereditary cancers (Invitae®; CA, USA) (These genes are listed in the supplementary materials). Variant classification is a systematic process for assessing the evidence gathered during variant review and applying a formal variant classification based on the recommendations from the American College of Medical Genetics (ACMG) (Nykamp et al., 2017). However, all results were re-checked and analyzed by geneticist at our institute. If germline mutation was detected, genetic counseling was provided to both patients and their relatives, if they agreed, and comprehensive cancer surveillance was provided.

Statistical analysis was carried out using SPSS version 22.0 (IBM Corporation, Armonk, New York, USA). Categorical variables were calculated using a Chi-square or Fisher Exact test. Continuous variables were tested using a Student t-test. Statistical significance was determined as p-value less than 0.05.

## Results

A total of 166 endometrial cancer patients were included. Demographic data and pathological data are shown in Table 1. The mean age was 57.1 + 10.9 years (range 20-83 years). Thirty-two patients (19.3%) were diagnosed at age younger than 50 years and 94 patients (56.6%) were younger than 60 years. Forty patients (24.1%) met the revised Bethesda Guidelines. Nine patients (5.4%) had a family history of Lynch syndrome-related cancers, and 11 patients (6.6%) had synchronous endometrial and ovarian or colon cancers. Stage I was found in 73.5% of the patients. Stage II, III, and IV were found in 7.2%, 16.9%, and 2.4% of the patients, respectively. 

Among 166 patients, 58 (34.9%) had one or more MMR deficiencies: MLH1 and PMS2 in 42 patients (25.3%), MSH2 and MSH6 in 11 patients (6.6%), and MSH6 in 5 patients (3.0%). There were no significant differences in clinicopathological characteristics between patients with and without MMR deficiency. Of the 40 patients who met the revised Bethesda Guidelines, 19 patients (47.5%) had MMR deficiency. In contrast, MMR deficiency was found in 39 of the 126 patients (31.0%) who did not meet the revised Bethesda Guidelines. Using the clinical criteria of diagnosis at age below 50 years and synchronous cancers, MMR deficiency was found in less than half of the patients; 43.8% (14/32 patients) and 45.5% (5/11 patients), respectively. In patients aged over 60 years, MMR deficiency was detected in 19 of 65 patients (29.3%): MLH1/PMS2 (n=15), MSH2/MSH6 (n=3), and MSH6 (n=1). 

Germline testing was performed in 27 of the 58 MMR deficient patients (46.6%). Twenty-four patients were lost to follow-up or could not be contacted, 4 patients denied testing, and 3 patients died. Of the 27 patients, MMR deficiencies included: MLH1/PMS2 in 16 patients (59.3%), MSH2/MSH6 in 7 patients (25.9%), and MSH6 in 4 patients (14.8%). Five of 27 patients (18.5%) had germline MMR mutations, which included 2 patients with *MSH6* [c.2295C>A (p.Cys765*) and c.2239delC (p.Leu747*)], 2 patients with *PMS2* [c.811G>A (p.Gly271Ser) and c.2404C>T (p.Arg802*)], and 1 patient with *MLH1* mutation [(c.109G>A (p.Glu37Lys)].* MSH6* mutations were found in 1 patient with loss expression of MSH2/MSH6 and 1 patient with loss of expression of MSH6. Among 3 patients with loss of expression of MLH1/PMS2, the PMS2 mutation was found in 2 patients and MLH1 mutation was found in 1 patient (Table 2).

Incidental germline mutations in other genes were detected in 3 patients including; 1 *BRCA1 *[c.1265_1266dupAT (p.Ser4231lefs*8)], 1 *BARD1* [c.808G>T (p.Glu270*), and 1 *PTEN* [c.493-2A>G (Splice acceptor)]. All these 3 patients were younger than 60 years and had loss expression of MLH1 and PMS2 deficiencies. Only the patient with *BRCA1* mutation had a family history of gynecologic cancer with unknown origin. The patient with PTEN mutation had co-incident findings with pathognomonic skin lesions of Cowden syndrome, such as papillomatous papules on the face and mucous membranes and multinodular goiter. All endometrial cancer patients with germline MMR mutations, including 3 other gene mutations, were younger than 60 years. 

Among 40 patients who met the revised Bethesda Guidelines, 21 had MMR proficiency (52.5%). Most patients with MMR proficiency (61.9%) met the criteria of age below 50 years, 3 patients (14.3%) had a family history of cancers, and 5 patients (23.8%) had synchronous cancers. Germline testing was performed in 8 of 21 patients (38.1%) with MMR proficiency but met the revised Bethesda Guidelines. None could detect germline MMR mutation. Based on the finding that germline MMR mutations were found in 18.5% (5/27) of the patients with MMR deficiencies, the predictive prevalence of germline MMR mutation in all endometrial cancer patients in this study was 6.5%. Germline MMR mutation was diagnosed in 6.3% of patients younger than 50 years (2/32 patients) and 4.3% of patients between 50-60 years (3/69 patients). The mean age of the patients with germline MMR mutation was significantly younger than sporadic patients (50 versus 56.7 years, p=0.03). Both patients with *MSH6* mutation were older than 50 years. Moreover, 40% of the patients who had germline MMR mutation did not meet Bethesda Guidelines. (Table 3) Seven of the 19 patients aged over 60 years with MMR deficiency underwent germline testing. The MMR deficiencies included: MLH1/PMS2 in 5 of 15 patients (33.3%), MSH2/MSH6 in 1 of 3 patients (33.3%), and MSH6 in 1 of 1 patient (100%). However, no germline mutation was detected in any patients aged over 60 years.

**Table 1:** Demographic Data and Pathological Findings

	All cases (N=166)	MMR deficiency (N=58)	MMR proficiency (N=108)	p-value
Age, mean *±* SD (years)	57.1 *±* 10.9 (range 20-83)	55.8 *±* 11.1 (range 30-79)	57.7 *±* 10.8 (range 20-83)	0.27
Age < 40 years, n (%)	10 (6)	3 (5.2)	7 (6.5)	1.00
Age < 50 years, n (%)	32 (19.3)	14 (24.1)	18 (16.7)	0.30
Age < 60 years, n (%)	94 (56.6)	38 (65.5)	56 (51.9)	0.10
Nulliparous, n (%)	70 (42.2)	22 (37.9)	48 (44.4)	0.51
Menopause, n (%)	108 (65.5)	35 (61.4)	73 (67.6)	0.39
BMI, mean *±* SD (kg/m^2^)	26.9 *±* 6.5 (range 16-49.4)	27.6 *±* 6.4 (range 18.3-43.7)	26.5 *±* 6.5 (range 16-49.4)	0.32
Obesity (BMI>30kg/m^2^), n (%)	41 (24.7)	15 (25.9)	26 (24.1)	0.85
Diabetes mellitus	29 (17.5)	7 (12.1)	22 (20.4)	0.20
Polycystic ovarian syndrome	2 (1.2)	1 (1.7)	1 (0.9)	1.00
Family history of cancers, n (%)	9 (5.4)	6 (10.3)	3 (2.8)	0.07
Bethesda guidelines, n (%)	40 (24.1)	19 (32.8)	21 (19.4)	0.06
Stage, n(%)				
I	122 (73.5)	46 (79.3)	76 (70.4)	0.61
II	12 (7.2)	4 (6.9)	8 (7.4)	
III	28 (16.9)	7 (12.1)	21 (19.4)	
IV	4 (2.4)	1 (1.7)	3 (2.8)	
Histology, n (%)				
Endometrioid carcinoma	156 (94)	56 (96.6)	100 (92.6)	0.22
Mixed adenocarcinoma	6 (3.6)	1 (1.7)	5 (4.6)	
Papillary serous carcinoma	1 (0.6)	1 (1.7)	0	
Carcinosarcoma	3 (1.8)	0	3 (2.8)	
Tumor grade, n (%)				
G1	91 (54.8)	29 (50)	62 (57.4)	0.34
G2	32 (19.3)	10 (17.2)	22 (20.4)	
G3	43 (25.9)	19 (33.8)	24 (22.2)	
Myometrial invasion, n (%)				
<50%	103 (62)	34 (58.6)	69 (63.9)	0.51
>= 50%	63 (38)	24 (41.4)	39 (36.1)	
Lymphovascular space invasion (N=110), n (%)	46 (41.8)	17/36 (47.2)	29/74 (39.2)	0.52
Lower uterine segment involvement, n (%)	103 (62)	37 (63.8)	66 (61.1)	0.87
Pelvic node metastasis (N=151), n (%)	18 (11.9)	4/54 (7.4)	14/97 (14.4)	0.30
Paraaortic node metastasis (N=85), n (%)	5 (5.9)	2/34 (5.9)	3/51 (5.9)	1.00
Synchronous endometrial and ovarian and/or colon cancers, n (%)	11 (6.6)	5 (8.6)	6 (5.6)	0.52
Adjuvant treatment, n (%)				
None	68 (41)	20 (34.5)	48 (44.4)	0.52
Pelvic radiation with brachytherapy	30 (18.1)	13 (22.4)	17 (15.7)	
Concurrent chemoradiation	24 (14.5)	8 (13.8)	16 (14.8)	
Brachytherapy	22 (13.3)	10 (17.2)	12 (11.1)	
Pelvic radiation then adjuvant chemotherapy	1 (0.6)	1 (1.7)	0	
Chemotherapy then pelvic radiation	4 (2.4)	1 (1.7)	3 (2.8)	
Chemotherapy	17 (10.2)	5 (8.6)	12 (11.1)	

**Table 2:** Details of Endometrial Cancer Patients with Germline Mutation

Age (years)	MMR deficiency by IHC	Gene	Nucleotide change	Variant classification	Family history of cancer	Synchronous cancers
47	MLH1+PMS2	*PMS2*	c.2404C>T	Pathogenic		-
46	MLH1+PMS2	*MLH1*	c.109G>A	Likely pathogenic	Colon cancer (father, grandfather, uncle)	Endometrial and ovarian cancer
51	MLH1+PMS2	*PMS2*	c.811G>A	Likely pathogenic	Colon cancer (Brother)	Endometrial and colon cancer
52	MSH2+MSH6	*MSH6*	c.2295C>A	Pathogenic	-	-
54	MSH6	*MSH6*	c.2239delC	Pathogenic	-	-
33	MLH1+PMS2	*BRCA1*	c.1265_1266dupAT	Pathogenic	Gynecologic cancer, unknown origin (grandmother)	-
51	MLH1+PMS2	*PTEN*	c.493-2A>G	Pathogenic	-	-
52	MLH1+PMS2	*BARD1*	c.808G>T	Pathogenic	-	-

**Table 3:** Clinical Characteristics between Endometrial Cancer Patients with and without Germline MMR Mutation

Characteristics	Germline MMR mutation (N=5)	No germline MMR mutation (N=19)	p-value
Mean age, *±* SD (years)	50.0 *±* 3.4	56.7 *±* 10.8	0.03
Family history of cancers, n(%)	2 (40)	2 (10.5)	0.18
Synchronous cancers, n(%)	2 (40)	3 (15.8)	0.27
Bethesda guidelines, n(%)	3 (60)	8 (42.1)	0.63

*Three patients with non-MMR germline mutation were excluded.

## Discussion

The loss of MMR expression in this study was found in 58 of 164 patients (35%), in agreement with previously published studies: 25% to 35% (Egoavil et al., 2013; Moline et al., 2013; Long et al., 2014; Rubio et al., 2016). Germline MMR mutations were detected in 5 of 27 patients (18.5%) including MSH6 (n=2), PMS2 (n=2), and MLH1 mutations (n=1). Among 5 patients with germline MMR mutation, 3 patients (60%) met the revised Bethesda Guidelines; all patients with germline mutation were younger than 60 years. Early onset of cancer is common in all hereditary cancer syndromes. The mean age of endometrial cancer patients with Lynch syndrome is 47-49 years (Broaddus et al., 2006) except those with *MSH6 *mutation, the mean age is over 50 years (Rubio et al., 2016). In contrast, the mean age in sporadic endometrial cancer patients is 61-63 years (Leenen et al., 2012; Egoavil et al., 2013; McConechy et al., 2015). The current study showed the mean age of 50 years in patients with Lynch syndrome versus 57 years in sporadic patients. The incidence of Lynch syndrome in endometrial cancer patients diagnosed before 50 years old (5-13%) is higher than patients between 50 and 60 years (3-5%) (Committee on Practice Bulletins-Gynecology and the Society of Gynecolgoic Oncology, 2014). This is in agreement with our study where rates are 6.3% and 4.3%, respectively. 

Incidental germline mutations in other genes were detected in 3 patients (1 *BRCA1*, 1 *PTEN*, and 1 *BARD1*). Among these three genes, only PTEN mutation associates with increased risk of endometrial cancer. PTEN mutation is a cause of Cowden syndrome, which is an autosomal dominant cancer syndrome and increases risk of multiple early-onset cancers such as breast, thyroid, endometrial and colon cancer. The lifetime risk of endometrial cancer increases from 3% to 40-60% (Pilarski et al., 2013). The patient with PTEN mutation in this study also met the clinical criteria of Cowden syndrome, such as papillomatous papules on the face and mucous membranes and multinodular goiter. The association of *BRCA* mutation and increased risk of endometrial cancer is controversial. However, *BRCA1* mutation may increase risk for serous subtype of endometrial cancer but not endometrioid subtype (Shu et al., 2016).

The revised Bethesda Guidelines have been used to identify patients at risk for Lynch syndrome. Although, the guidelines have high sensitivity, they have low specificity for identifying potential Lynch syndrome patients ( 2014). As a result, there are not only high false positive rates, but also increased costs of genetic testing. Immunohistochemistry for MMR proteins are a more cost-effective screening approach and highly sensitive to identify Lynch syndrome patients (Mills et al., 2014). One large population-based study reported that up to 70% of endometrial cancer patients with Lynch syndrome did not meet these clinical criteria and 60% were diagnosed aged above 50 years (Hampel et al., 2006). About 40% of patients with germline MMR mutation in the current study did not meet the Bethesda Guidelines, similar to rates reported in a previous study (Mills et al., 2014). 

The Joint ACOG/SGO recommended Lynch syndrome screening in tumor tissue with microsatellite instability and/or MMR immunohistochemistry. If high microsatellite instability and/or loss of MMR expression are detected, germline MMR testing should be considered (Randall et al., 2017). The microsatellite instability and immunohistochemistry analyses have high sensitivity and specificity to screen patients at risk of Lynch syndrome. In addition, both techniques have high concordance (93-100%) (Walsh et al., 2008; Leenen et al., 2012; McConechy et al., 2015). MMR immunohistochemistry can be used alone or in conjunction with microsatellite instability (Hampel et al., 2006). In general, immunohistochemistry is less expensive, more cost-effective, and widely available (Stelloo et al., 2017). On the other hand, the major limitation of microsatellite instability testing is less accuracy in identifying endometrial cancer patients with the *MSH6* mutation. Low or no microsatellite instability is commonly found in the *MSH6* mutation carriers. The *MSH6* mutation is important in Lynch syndrome as it has a 5-fold increased risk of endometrial cancer when compared with colorectal cancer (Hampel et al., 2006; McConechy et al., 2015). Therefore, the MMR immunohistochemistry was used in the current study as an initial screening tool for germline MMR mutation. 

The loss of MLH1/PMS2 expression was the most common MMR deficiency in this study (25.3%). Although 10-20% of endometrial cancer patients had loss of MLH/*PMS2* expression, very few had germline *MLH1* or PMS2 mutations (Mills et al., 2014). Therefore, the loss of MLH1/PMS2 expression, including high microsatellite instability, may not be associated with a germline MMR mutation. It usually occurs in sporadic endometrial cancer patients who have *MLH1* promoter hypermethylation or somatic MMR mutation (Berends et al., 2003). The PCR for *MLH1* promoter methylation was not performed in 42 tumor specimens with loss expression of MLH1 due to unavailability in our institute. Therefore, the number of true candidates for further germline testing should be lower. In this study, the loss of MLH1/PMS2 expression was found more frequently in patients over 60 years old than the other MMR proteins. However, no germline MMR mutation was found in this age group. Most of these patients should be diagnosed as sporadic cases and could be explained by the occurrence of *MLH1 *hypermethylation or somatic MMR mutation. 

Lynch syndrome screening in endometrial cancer patients is crucial to identify which patients should be offered genetic counseling and genetic testing in order to prevent further Lynch syndrome-related cancers. Synchronous or metachronous cancers are common in Lynch syndrome patients. Although, colorectal cancer is the most common Lynch syndrome-related cancer, endometrial or ovarian cancer was the first diagnosed cancer in nearly 50% MMR carrier women. The presence of gynecologic cancers was 11 years (median duration) before colorectal cancers (Lu et al., 2005). Twenty-year cumulative risks of cancers after endometrial cancer diagnosis has been reported including, 48% risk for colorectal cancer, 11% for kidney and ureter cancer, 9% for bladder cancer, and 11% for breast cancer (Win et al., 2013). Intensive cancer surveillance with risk reducing surgeries should be considered. Additionally, a risk assessment should be offered to all family members to reduce cancer-related risk. Furthermore, MMR dysfunction either MMR deficiency by immunohistochemistry or MSI-high is one prognostic factor and indicates candidates for novel treatments with immune checkpoint inhibitors (Green et al., 2020).

Major drawback in this study was endometrial tumor tissues were derived from only one institute. All endometrial cancer patients did not perform germline testing. Genetic testing was done in nearly half of patients with MMR deficiency and almost 40% of MMR proficient patients who met the revised Bethesda Guidelines. A multicenter trial with a larger number of endometrial cancer patients is required to demonstrate the incidence of germline MMR mutation in the Thai population. 

In conclusion, Lynch syndrome screening with MMR immunohistochemistry should be considered in endometrial cancer patients, regardless of personal or family history of Lynch syndrome-related cancers. Further genetic counseling and testing should be offered to patients at risk for Lynch syndrome and their relatives in order to diagnose and prevent other Lynch syndrome-related cancers.

## Author Contribution Statement

TM designed the research study, collected, analyzed and interpreted the data and wrote the manuscript; CA and ST performed the histological examination and interpreted the immunohistochemistry study; PP provided genetic counseling, interpreted the genetic database. All authors read and approved the final manuscript.
